# Expression of active human sialyltransferase ST6GalNAcI in *Escherichia coli*

**DOI:** 10.1186/1475-2859-8-50

**Published:** 2009-09-30

**Authors:** Georgios Skretas, Sean Carroll, Shawn DeFrees, Marc F Schwartz, Karl F Johnson, George Georgiou

**Affiliations:** 1 Department of Chemical Engineering, University of Texas at Austin, Austin, TX 78712, USA; 2 Department of Biomedical Engineering, University of Texas at Austin, Austin, TX 78712, USA; 3 Section of Microbiology and Molecular Genetics, University of Texas at Austin, Austin, TX 78712, USA; 4 Institute for Cellular and Molecular Biology, University of Texas at Austin, Austin, TX 78712, USA; 5 Neose Technologies Inc, 102 Rock Road, Horsham, PA, 19044, USA

## Abstract

**Background:**

The presence of terminal, surface-exposed sialic acid moieties can greatly enhance the *in vivo *half-life of glycosylated biopharmaceuticals and improve their therapeutic efficacy. Complete and homogeneous sialylation of glycoproteins can be efficiently performed enzymically *in vitro *but this process requires large amounts of catalytically active sialyltransferases. Furthermore, standard microbial hosts used for large-scale production of recombinant enzymes can only produce small quantities of glycosyltransferases of animal origin, which lack catalytic activity.

**Results and conclusion:**

In this work, we have expressed the human sialyltransferase ST6GalNAc I (ST6), an enzyme that sialylates O-linked glycoproteins, in *Escherichia coli *cells. We observed that wild-type bacterial cells are able to produce only very small amounts of soluble ST6 enzyme. We have found, however, that engineered bacterial strains which possess certain types of oxidative cytoplasm or which co-express the molecular chaperones/co-chaperones trigger factor, DnaK/DnaJ, GroEL/GroES, and Skp, can produce greatly enhanced amounts of soluble ST6. Furthermore, we have developed a novel high-throughput assay for the detection of sialyltransferase activity and used it to demonstrate that the bacterially expressed ST6 enzyme is active and able to transfer sialic acid onto a desialylated O-glycoprotein, bovine submaxillary mucin. To the best of our knowledge, this is the first example of expression of active human sialyltransferase in bacteria. This system may be used as a starting point for the evolution of sialyltransferases with better expression characteristics or altered donor/acceptor specificities.

## Background

The covalent attachment of oligosaccharides on peptides and proteins (protein glycosylation) is one of the most complex and frequent post-translational modifications in eukaryotes [1]. More than 30% of all therapeutic proteins are glycosylated (glycoproteins) and their folding, biological activity, biodistribution and pharmacological efficacy is critically dependent on the attachment of the correct glycan structure [1]. Most of the widely used bacterial expression hosts, such as *Escherichia coli*, cannot perform protein glycosylation [1].  Although eukaryotic hosts, such as yeast, insect, and non-human mammalian cell lines are capable of protein glycosylation, they introduce non-native glycosylation patterns that can result in undesired effects, including decreased biological potency and immunogenicity [1]. While animal cells have been engineered to produce proteins containing glycans that resemble more those found in humans, an ensemble of partially glycosylated forms is also synthesized and these have to be removed during downstream processing [1].

One of the most important examples of the influence of glycan structure on pharmacological properties of therapeutic proteins is the strong dependence of the serum half-life of a glycoprotein on the presence of sialic acid moieties attached to the terminal site of its glycans [2]. For example, recombinant human erythropoietin (EPO) normally has a plasma half-life of more than five hours in rodents [3]. Removal of the terminal sialic acid moieties (desialylation) prior to administration reduces EPO's half-life dramatically to less than 2 minutes [3]. It has been shown that the *in vivo *half-life and biological (hematopoietic) activity of EPO is directly proportional to the number of sialic acid moieties attached to its four naturally occurring glycosylation sites [4]. Site-specific incorporation of additional glycosylation sites into the protein's sequence and the resulting increase in the number of attached sialic acids per molecule has been shown to increase the half-life of EPO [5].

Due to its biological importance, the FDA has mandated the close monitoring of sialic acid content in therapeutic proteins [2]. Recently, glycoengineered mammalian, insect and yeast cell lines have been developed with the ability to achieve enhanced sialylation levels for recombinant glycoproteins [2]. A promising alternative approach for producing fully sialylated proteins is to isolate the recombinantly produced polypeptide and append the sialic acid moiety *in vitro *using purified sialyltransefases and activated sialic acid donors (GlycoAdvance™ system, Neose Technologies Inc.). *In vitro *sialylation, however, is predicated on the availability of large quantities of the glycosyltrasferase enzymes. However, extensive studies have shown that the expression of mammalian glycosyltransferases in bacteria or lower eukaryotes typically results in very low yields of soluble and active enzyme [6]. In particular, there have been no reports of active human sialyltransferase expression in bacteria.

In this work, we report the expression of the human sialyltransferase ST6GalNAcI (ST6), an enzyme that sialylates O-linked glycoproteins, in *E. coli *cells. We are interested in studying O-linked glycosylated proteins because very little is known about O-glycosylation compared to N-glycosylation [7]. While wild-type bacterial cells were found to produce very small amounts of soluble ST6, engineered strains of *E. coli *which possess certain types of oxidative cytoplasm or which co-express specific molecular chaperones, can result in significant yields of ST6. Furthermore, using a novel high-throughput assay for the detection of sialyltransferase activity, we show that the bacterially produced ST6 is active and capable of catalyzing the transfer of sialic acid onto a desialylated O-glycoprotein, bovine submaxillary mucin.

## Results

### Expression of ST6 in *E. coli *strains having an oxidizing cytoplasm

ST6 catalyzes the transfer of N-acetylneuraminic acid (Neu5Ac - the most common type of sialic acid in higher animals) from the sugar donor CMP-Neu5Ac (or CMP-SA) onto a terminal β-D-galactopyranosyl (Gal) residue of an O-linked glycoprotein to generate an α 2-6 linkage [8]. Like other sialyltransferases, ST6 is a type II transmembrane glycoprotein, comprised of a short N-terminal cytosolic tail, a hydrophobic signal-anchor sequence that is embedded in the membrane, a so-called "stem" region, and a long C-terminal catalytic domain that is exposed to the lumen of the Golgi apparatus. In general, the catalytic domains of sialyltransferases do not require the other three domains in order to maintain enzymic activity [8]. A codon-optimized gene encoding the catalytic domain of the human ST6 (amino acids Lys36-end, coST6) was cloned into pTrc99a downstream from the *tac *promoter. To facilitate immunodetection and protein purification, a FLAG tag and an octahistidine tag were fused at the N- and C- termini, respectively (Table 1). In addition, coST6 was cloned into the high-copy number plasmid pCWin2MBP [9] to generate the vector pCWin2MBP-ST6. pCWin2MBP-ST6 expresses an N-terminal fusion of ST6 with the *E. coli *maltose-binding protein (MBP-ST6) under the control of a dual *tac *promoter.

**Table 1:** Plasmids used in this work

**Plasmid**	**Protein expressed**	**Antibiotic Marker**	**Origin of replication**	**Source**
**pTrcST6**	FLAG-ST6-His_8_	AmpR	ColE1	This work
**pCWinMBP-ST6**	MBP-ST6	KanR	ColE1	This work
**pBAD33**	Empty vector	CmR	ACYC	Guzman et al.^1^
**pBADtig**	Trigger Factor	CmR	ACYC	Ref. [17]
**pBADΔssDsbA**	Signal sequence-less DsbA (ΔssDsbA)	CmR	ACYC	Ref. [11]
**pBADΔssDsbC**	Signal sequence-less DsbC (ΔssDsbC)	CmR	ACYC	Ref. [11]
**pAKJ**	DnaK/DnaJ	CmR	ACYC	Perez-Perez et al.^2^
**pAG**	GroEL/GroES	CmR	ACYC	Perez-Perez et al.^2^
**pAS**	Signal sequence-less Skp (ΔssSkp)	CmR	ACYC	Ref. [17]

1. Guzman LM, Belin D, Carson MJ, Beckwith J: **Tight regulation, modulation, and high-level expression by vectors containing the arabinose PBAD promoter**. *J Bacteriol *1995, **177**(14):4121-4130.

2. Perez-Perez J, Martinez-Caja C, Barbero JL, Gutierrez J: **DnaK/DnaJ supplementation improves the periplasmic production of human granulocyte-colony stimulating factor in Escherichia coli**. *Biochem Biophys Res Commun *1995, **210**(2):524-529.

**Figure 1:**
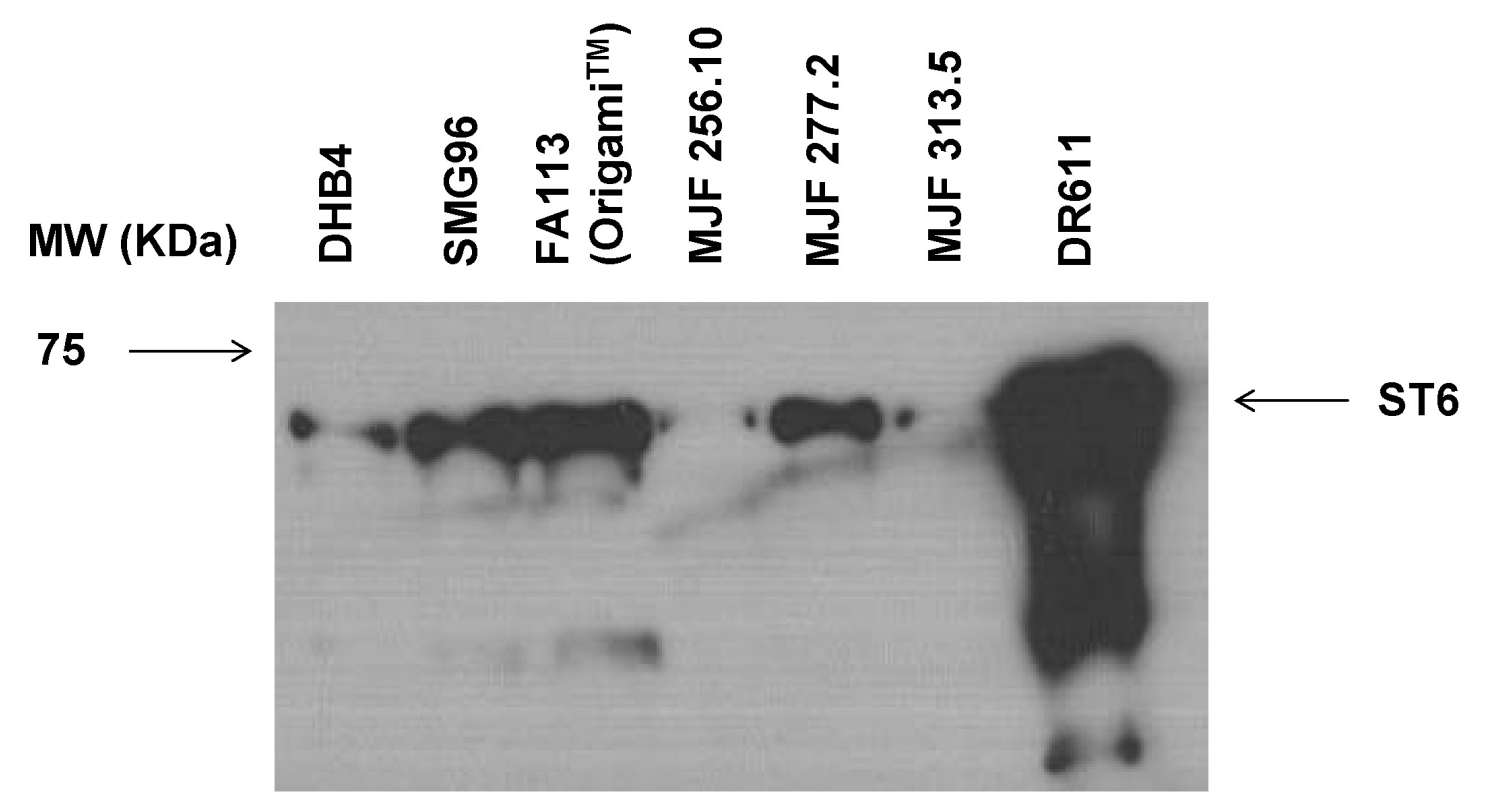
**Comparison of the production of soluble ST6 in wild-type (DHB4) and *E. coli *strains with oxidizing cytoplasm by Western blotting**. Proteins were probed with an anti-polyHis antibody. Lanes contain equal volumes of bacterial culture. MW: molecular weight

The catalytic domain of human sialyltransferases contains two highly conserved cysteine residues that form a disulfide bond which is required for proper folding and activity [10]. The cytoplasmic space of wild-type *E. coli *cells is normally maintained in a reduced state that precludes the formation of disulfide bonds via the action of the thioredoxin and glutaredoxin/glutathione enzyme systems. Mutant strains defective in glutathione reductase (*gor*) or glutathione synthetase (*gshA*) together with thioredoxin reductase (*trxB*) render the cytoplasm oxidizing but are unable to reduce ribonucleotides and therefore cannot grow in the absence of exogenous reductant, such as DTT. However, suppressor mutations in the gene *ahpC *which encodes the peroxiredoxin AhpC, allow the channeling of electrons onto the enzyme ribonulceotide reductase enabling the cells to grow in the absence of DTT. In such strains, exposed protein cysteines become readily oxidized in a process that is catalyzed by thioredoxins, in a reversal of their physiological function, resulting in the formation of disulfide bonds. A number of heterologous multidisulfide bonded proteins have been produced in the cytoplasm of *E. coli *FA113 cells (trxB gor ahpC*) or Origami™ at high yields [11]. Additionally, it was recently shown that bacterial strains with different mutations in the thioredoxin/thioredoxin reductase and glutaredoxin/glutathione reductase genes and containing different suppressor mutations in alleles of *ahpC*, display dramatic differences in the kinetics of cysteine oxidation in the cytoplasm and in the yield of correctly folded proteins [12]. We compared the expression of soluble ST6 in a variety of *E. coli *strains with oxidizing cytoplasm (Table 2). A large increase in the amount of soluble ST6 protein was observed in the *E. coli *strains SMG96 (Δ*gor *ΔtrxB ahpC*), FA113 (*gor*522.miniTn10 Δ*trxB*::Kan^R ^ahpC*), MJF277.2 (Δ*gshB*::Kan^R ^ΔtrxB ahpC*), and DR611 (Δ*trxA *Δ*trxC trxB*::Kan^R ^*gor*522:Tn10 *ahpC *T104P/G141C) relative to the parental strain DHB4 (Figure 1). Among these, *E. coli *DR611 cells were able to produce dramatically increased amounts of soluble ST6.

**Table 2:** Investigated *E. coli *Strains with Oxidizing Cytoplasmic Space

**Strain**	**Genotype**	**Source**
**DHB4 (parental)**	Δ*(ara-leu)7697 araD139 ΔlacX74 galE galK rpsL phoR* *Δ(phoA)PvuII ΔmalF3 thi/F Δlac-pro lacIq*	Boyd et al.^1^
**SMG96**	Δ*gor *ΔtrxB ahpC*	Ref. [12]
**FA113**	*gor*522.miniTn10 (Tet^R^) Δ*trxB*::Kan^R ^ahpC*	Ref. [11]
**Origami**™**2**	*gor*522:miniTn10 *trxB*::Strep^R^, Tet^R ^ahpC*	Novagen
**MJF256.10**	Δ*gshA*::Kan^R^Δ*trxB*::Cm^R ^*ahpC *V164G	Ref. [12]
**MJF277.2**	Δ*gshB*::Kan^R^ΔtrxB ahpC*	Ref. [12]
**MJF313.5**	Δ*gshA *Δ*trxB*::Cm^R ^*ahpC *E171 stop	Ref. [12]
**DR611**	Δ*trxA *Δ*trxC trxB*::Kan^R ^*gor*522:Tn10 *ahpC *T104P/G141C	J. Beckwith

1. Boyd D, Manoil C, Beckwith J: **Determinants of membrane protein topology**. *Proc Natl Acad Sci USA *1987, **84**(23):8525-8529.

### Co-expression of molecular chaperones enhances the production of soluble ST6

**Figure 2:**
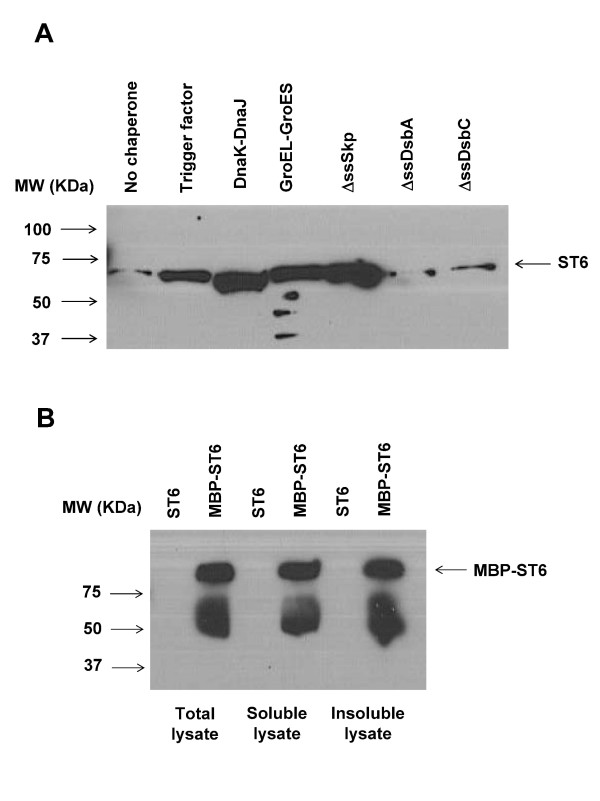
**(A). Comparison of the production of soluble ST6 in Origami™2 cells in the presence of different over-expressed molecular chaperones by Western blotting**. **(B)**. Comparison of the production of ST6 (expected molecular weight (MW) ~ 65 KDa) and MBP-ST6 (expected MW ~ 104 KDa) in different fractions of Origami™2 cells by Western blotting. All lanes show similar ST6 band intensities because of signal saturation. Without fusion to MBP, a band corresponding to ST6 could not be detected at the tested short exposure times. Lanes contain equal volumes of bacterial culture. Proteins were probed with an anti-polyHis antibody.

Extensive studies have shown that the solubility of heterologous proteins in bacteria can be increased greatly by co-expression of molecular chaperones [13] or by fusion to a highly soluble protein partner, such as MBP [14]. We tested the effect of the chaperones/co-chaperones trigger factor, DnaK/DnaJ, GroEL/GroES, a cytoplasmically expressed variant of the periplasmic chaperone/peptidyl-prolyl isomerase Skp which lacks its signaling sequence (ΔssSkp), or similarly expressed disulfide oxidoreductase DsbA (ΔssDsbA) or disulfide bond isomerase DsbC (ΔssDsbC) (Table 1). Co-expression of trigger factor, DnaK/DnaJ, GroEL/GroES, and ΔssSkp resulted in a significant increase in the amount of soluble ST6 in Origami 2 cells (Figure 2A). However, chaperone co-expression did not result in a further increase in the soluble yield of ST6 in DR611, which, as discussed above, already accumulates high levels of soluble protein (data not shown).

Fusion of the ST6 enzyme to MBP also resulted in a marked increase in the amount of soluble ST6 in Origami 2 cells (Figure 2B), as well as in the DR611 strain (data not shown). The co-expression of molecular chaperones had similar enhancing effects on the production of soluble MBP-ST6 as in the case of MBP-free ST6 (data not shown).

It must be mentioned that in all the cases where ST6 production was tested (different strains, molecular chaperones, fusion partners) by Western blotting, the presence of lower molecular weight bands was observed. These bands indicate protein degradation, presumably due to the low stability of the ST6 enzyme or due to proteolytic degradation. Degradation, however, appears to be limited as can be deduced by comparing the intensity of the band corresponding to full-length enzyme relative to the putative lower-molecular-weight proteolytic products and should not interfere significantly with the production of the ST6 enzyme in the described strains.

### Sialyltransferase activity

**Figure 3:**
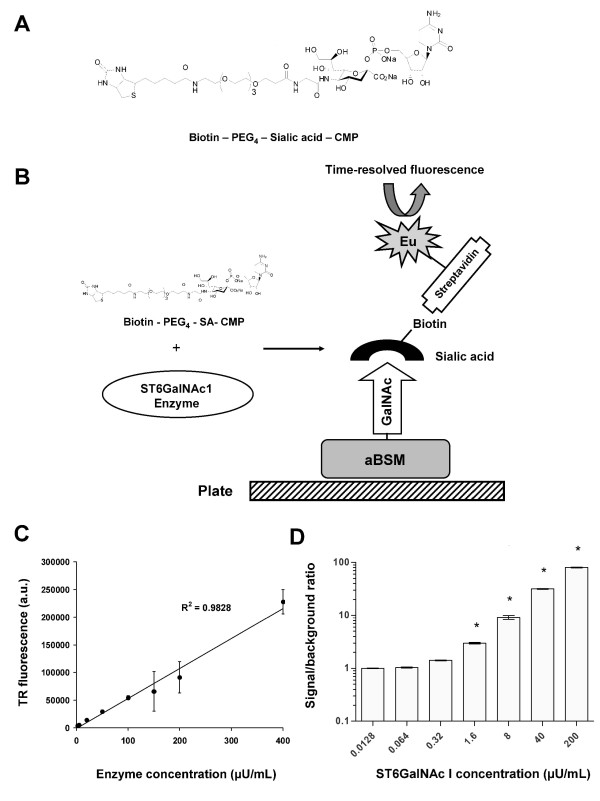
**(A). Chemical structure of the synthetic sugar donor substrate CMP-sialic acid-PEG_4_-biotin utilized in our high-throughput assay for sialyltransferase activity**. **(B)**. Schematic of the developed assay for sialyltransferase activity. 96- or 384-well plates are coated initially with asialo bovine submaxillary mucin (aBSM). aBSM carries exposed terminal GalNAc moieties. Active ST6GalNAcI catalyzes the transfer of sialic acid- PEG_4_-biotin from the sugar donor substrate CMP-sialic acid-PEG_4_-biotin onto aBSM and immobilizes biotin on the plate. Biotinylation can be subsequently detected with europium (Eu)-labeled streptavidin and time-resolved fluorescence. **(C)**. Time-resolved (TR) fluorescence counts plotted against different concentrations of chicken ST6 expressed in Sf9 insect cells (positive control enzyme). **(D)**. Detection limit of the sialyltransferase assay. Calculated signal-to-background (no enzyme control) ratios for different concentrations of chicken ST6 expressed in Sf9 insect cells. Asterisks indicate enzyme concentrations that exhibited enzymic activity which was statistically different from the no enzyme control (Dunnett's MCT, p < 0.01). Experiments were carried out in triplicate and the error bars correspond to one standard deviation from the mean values. a.u.: arbitrary units; U: unit of sialyltransferase activity.

In order to evaluate the activity of the produced ST6 enzyme in our engineered *E. coli *strains, we developed a non-radioactive high-throughput assay for sialyltransferase activity. The assay utilizes a sialic acid donor (CMP-Neu5Ac or CMP-SA), which is tagged with a short polyethylene glycol (PEG_4_) spacer and biotin (CMP-SA-PEG_4_-biotin) for detection (Figure 3A). First, an appropriate acceptor protein, such as bovine submaxillary mucin lacking its terminal sialic acid moieties, is coated on a microtiter plate (Figure 3B). The acceptor is then incubated with the CMP-SA-PEG_4_-biotin sugar donor and the tested enzyme. Biotinylated mucin can be readily detected with europium-labeled streptavidin using time-resolved fluorescence (Figure 3B). ST6 produced in Sf9 insect cells was used to calibrate sialyltransferase activity (Figure 3C). The lower detection limit was determined to be 2 μU/mL (Figure 3D). The assay can be performed in 96- and 384-well microtiter plate format and at reaction volumes as low as 10 μL (data not shown).

**Figure 4:**
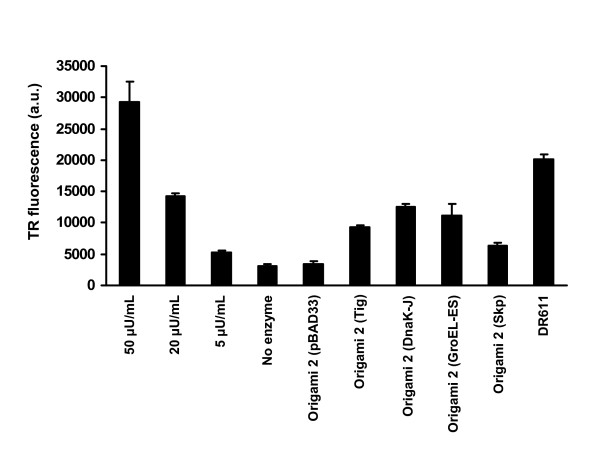
**Time-resolved (TR) fluorescence counts of different concentrations of chicken ST6 expressed in Sf9 insect cells (0, 5, 20 and 50 μU/mL reaction) and of clarified lysates of bacterial cells expressing MBP-ST6**. The bacterial strains used were DR611 and Origami 2 cells, without (pBAD33) or with co-expression of the molecular chaperones/co-chaperones trigger factor (Tig), DnaK/DnaJ (DnaK-J), GroEL/GroES (GroEL-ES), and ΔssSkp (Skp). Each sample of bacterial cell lysate contained equal number of cells. Experiments were carried out in replica triplicates and the error bars correspond to one standard deviation from the mean values. a.u.: arbitrary units; U: unit of sialyltransferase activity.

We found that even though Origami 2 cells produce appreciable amounts of soluble MBP-ST6, only a very low level of mucin sialylation activity could be detected (Figure 4). However, the co-expression of the molecular chaperones/co-chaperones trigger factor, DnaK/DnaJ, GroEL/GroES and ΔssSkp resulted in a significant increase in the yield of active ST6 (Figure 4). Importantly, the expression of MBP-ST6 in DR611 cells resulted in markedly enhanced levels of sialyltransferase activity (Figure 4), resulting in lysate activity of approximately 0.7 U/L of bacterial shake flask culture.

## Discussion

We found that similar to most other glycosyltransferases expressed in bacteria [6], expression of the human sialyltransferase ST6 in wild-type *E. coli *cells results in very low levels of soluble protein with essentially no catalytic activity. All human sialyltransferases contain an essential disulfide bond whose formation is strongly disfavored within the reducing cytoplasm of wild-type bacterial cells [10]. However, significant amounts of soluble human ST6 could be produced in *E. coli *strains having an oxidizing cytoplasmic space and, additionally, by either co-expressing molecular chaperones or using MBP fusions. Using a novel assay for sialyltransferase activity, we found that the ST6 enzyme produced in these engineered strains is catalytically active in the transfer of sialic acid onto a donor glycoprotein.

We showed that the overexpression of trigger factor, DnaK/DnaJ, GroEL/GroES, and ΔssSkp but not of enzymes involved in disulfide bond formation (ΔssDsbA and ΔssDsbC) markedly increased solubility in Origami 2 cells. Co-expression of trigger factor, DnaK/DnaJ, and GroEL/GroES has been found to be effective in enhancing the bacterial production of recombinant proteins in a number of previous studies [15]. The enhancement of ST6 production accompanying the co-expression of the normally periplasmic chaperone/peptidyl-prolyl isomerase Skp, however, is particularly interesting. Although Skp co-expression has been previously found to enhance the production of certain antibody fragments in *E. coli *[16,17] and to prevent aggregation of lysozyme [18], this protein is primarily a molecular chaperone for bacterial outer membrane proteins [19]. Our results in combination with previous studies [16-18], may indicate a more general substrate specificity for Skp. Indeed, very recent proteomics studies have shown that Skp does not only interact with bacterial outer membrane proteins, but also with a variety of periplasmic soluble proteins in *E. coli *[20].

The *E. coli *strain DR611, which we found to be the most effective for the production of soluble and catalytically active ST6, lacks all three components of the thioredoxin/thioredoxin reductase pathway (*trxA*, *trxB *and *trxC*), the first component of the glutathione/glutaredoxin pathway (*gor*), and carries a pair of suppressor mutations in *ahpC*. Unlike other oxidizing strains where the catalysis of disulfide bond formation in the cytoplasm is mediated by the thioredoxins, in this strain protein oxidation obviously occurs via a different mechanism. It is possible that the two substitutions allow AhpC to function as a reductase for glutathionylated glutaredoxins and supply reduced glutathione to the cell, in a fashion similar to the other identified *ahpC *suppressor mutations in Δ*trxB *Δ*gor *strains [21]. The presence of reduced glutathione together with oxidized glutathione that might accumulate as a consequence of aerobic growth in the absence of *gor *and *trxB*, could mediate the formation of a redox buffer that provides favorable kinetics for the formation of the disulfide bond in ST6, resulting in turn in greater solubility. Another possible reason for the ability of DR611 to produce markedly enhanced amounts of ST6 is that in a Δ*trxA *background, certain components of the protein quality control/degradation machinery of the *E. coli *cell may be activated or inactivated. Proteomic analysis has revealed that thioredoxin interacts with at least 80 different proteins in *E. coli*, a number of which are molecular chaperones, co-chaperones, chaperone-regulating proteins, and proteases [22].

Irrespective of the mechanism, the production of soluble and catalytically active ST6 in *E. coli *strains opens up new possibilities for large-scale production of sialyltransferases and possibly of other glycosyltransferases as well. Although the ST6 activity yield in bacteria was found to be considerably lower than in higher cells (0.7 U/L in this work vs. 40 U/L of chicken ST6 produced in insect cells [our unpublished results]), the combination of expression and screening systems described here can be used for the directed evolution of enzyme variants displaying either better expression characteristics or altered substrate specificity. Also, oxidizing strains and chaperone co-expression could be useful for the bacterial production of other mammalian sialyltransferases and other disulfide-bonded glycosyltransferases as well. Lastly, our results provide further support that engineered *E. coli *cells with different types of oxidative cytoplasmic spaces can have a profound effect on the amount of properly folded disulfide-bonded proteins which can be produced in the bacterial cytoplasm [12].

## Conclusion

We have expressed the human sialyltransferase ST6GalNAcI in *E. coli *cells and found that wild-type bacterial cells are able to produce only very small amounts of soluble ST6 enzyme, which is catalytically inactive. We have found, however, that engineered bacterial strains which possess certain types of oxidative cytoplasmic spaces or which co-express the molecular chaperones/co-chaperones trigger factor, DnaK/DnaJ, GroEL/GroES, and Skp, can produce greatly increased quantities of soluble ST6. By utilizing a novel high-throughput assay for the detection of sialyltransferase activity, we showed that our engineered bacterial strains produce human ST6 in an active form, which was able to transfer sialic acid onto a desialylated O-glycoprotein, bovine submaxillary mucin. To our knowledge, this is the first example of an active human sialytransferase produced in bacteria.

## Methods

### Strains

*E. coli *MC4100A cells [23] were used for plasmid constructions. Origami™2 cells were purchased from Novagen. The strains DHB4, SMG96, FA113, MJF256.10, MJF277.2, MJF313.5, and DR611 were a kind gift from Jon Beckwith.

### Plasmids

All restriction enzymes and other DNA processing enzymes were purchased from New England Biolabs. The nucleotide sequence encoding human ST6GalNAcI starting with Lys36 was obtained by PCR amplification of EST clones (Invitrogen), and cloned between the *BamH*I and *Xba*I sites of pCWin2-MBP to generate the vector pCWin2-MBP ST6GalNAcI [9]. pCWin2-MBP ST6GalNAcI expresses a fusion of the soluble catalytic domain of ST6GalNAcI with the *E. coli *maltose-binding protein attached to its N-terminus, under the control of a dual *tac *promoter. To improve bacterial expression, DNA from between the *BamH*I and *Stu*I sites was synthesized using optimal codon selection (DNA2.0), and re-cloned into the pCWin2-MBP ST6GalNAcI expression vector using standard techniques to generate pCWin2-MBP coST6GalNAcI. In addition, coST6GalNAcI was PCR-amplified from pCWin2-MBP coST6GalNAcI (pCWin2MBP-ST6) along with an N-terminal FLAG, an optimized ribosome-binding site, and a C-terminal octahistidine tag and inserted between the EcoRI and XbaI sites of the plasmid pTrc99a (GE Healthcare) to generate the vector pTrcST6. pTrcST6 expresses MBP-free ST6 under the control of the *tac *promoter.

### Protein Expression

*E. coli *cells freshly transformed with the appropriate expression vector were used for all ST6 production experiments. Single bacterial colonies were used to inoculate liquid LB cultures containing 100 μg/mL ampicillin or 50 μg/mL kanamycin, depending on the utilized ST6 expression vector. These saturated cultures were used with a 1:100 dilution to inoculate fresh LB cultures which were grown at 37°C to an optical density at 600 nm (OD_600_) of 0.5-0.7 with shaking. The temperature was then decreased to 25°C and after a temperature equilibration period of 5-10 min, protein expression was induced by the addition of 0.1 mM isopropyl-β-D-thiogalactopyranoside (IPTG) for approximately 5 h. When ST6 was co-expressed with molecular chaperones, cells were grown in a similar fashion, but the growth medium contained additionally 40 μg/mL chloramphenicol and 0.1% L-arabinose.

### Western Blotting

Western blotting was performed as described previously [24].

### Synthesis of CMP-SA-PEG4-biotin

Cytidine-5'-monophospho-N-glycylsialic acid disodium salt (GSC; 2.2 g, 3.3 mmoles) was dissolved in a mixture of water (30 mL) and anhydrous THF (50 mL) in a 500 mL single neck round bottom flask equipped with a magnetic stir bar. NHS-dPEG™ _4_Biotin (Quanta Biodesign; 2.0 g, 3.34 mmoles) was added and the resulting colorless clear solution was stirred at room temperature for three hours. The mixture was concentrated under reduced pressure, diluted with 400 mL of water, and filtered through a 0.45 micron membrane filter to provide 600 mL of diluted reaction mixture (conductivity of 1.08 mS/cm).

A Q-Sepharose Big Beads chromatography column (GE Healthcare; XK-26/60 column packed to a bed height of 29 cm, column volume of 690 mL) was converted to the bicarbonate counterion form using 3 column volumes of 1 N NaHCO_3 _and washed with 4 column volumes of water. A portion of the filtered reaction mixture (300 mL) was loaded on to the column. The column was then washed with 4 column volumes of water using a flow rate of 20 mL/min and with 2.5 column volumes of a solution of 40 mM NaHCO_3_. The product was then eluted using a linear gradient from 40 mM NaHCO_3 _to 80 mM NaHCO_3 _over two column volumes. The eluted product was pooled (1181 mL) according to the UV profile (274 nm) and the solution freeze dried providing a white solid. The solid was dissolved in 200 mL of water and slowly loaded onto a G25 column (GE Healthcare; XK50/100 packed to a bed height of 44.5 cm; a column volume of 873 mL) at 20 mL/min. The product eluted with 5 column volumes of water. The product was collected, freeze-dried and to yield 0.81 g of a white solid. The second portion of filtered reaction mixture was purified using both Q-sepharose and G25 as described. The combined total yield was 1.28 grams of white solid; ^1^H NMR (D_2_O, ppm) 7.98 (d, 1H), 6.12 (d, 1H), 5.98 (d, 1H), 4.73 (m, 1H), 4.34 (t, 1H), 4.31 (t, 1H), 4.23 (d, 3H), 4.18 (d, 1H), 4.12 (dt, 1H), 3.99 (m, 3H), 3.89 (t, 1H), 3.87 (d, 1H), 3.80 (t, 2H), 3.62 (m, 4H), 3.42 (d, 1H), 3.38 (t, 1H), 3.36 (m, 1H), 3.23 (dd, 1H), 2.62 (t, 2H), 2.48, (dd, 1H), 2.30 (d, 2H), 1.86 (m, 2H), 1.65 (m, 3H), 1.55 (m, 2H); MS (negative mode; Q-star) 794.8, 1117.9, 1140.0.

### Mucin desialylation

Purified bovine submaxillary mucin (BSM) (Sigma) dissolved in 0.1 N H_2_SO_4 _was heated to 100°C and the desialylation reaction was monitored in order to avoid precipitation. After approximately one hour, the reaction mixture was cooled at room temperature and neutralized by the addition of 0.1N NaOH. Tris was added to a final concentration of 0.05 M. The solution was subsequently filtered, dialyzed three times against water at 4°C, lyophilized, and then resuspended in water (25 mg/mL) and stored at -80°C.

### Sialylation activity assays

Low-fluorescence yellow DELFIA 96-well plates (Perkin-Elmer) were coated with 50 μL of a 20 μg/mL desialylated BSM (aBSM) solution in coating buffer (50 mM NaH_2_PO_4_, pH 7.2) at room temperature for 1-2 h with shaking. Following aBSM aspiration, the wells were blocked with 250 μL of a 0.05% gelatin solution in phosphate-buffered saline (PBS) for 1-2 h at room temperature with shaking. The blocked wells were washed three times with excess PBS containing 0.1% Tween-20 (PBST), and 25 μL of double-concentrated reaction buffer (50 mM BisTris pH 6.7, 5 mM MnCl_2_, 0.1% Tween-20, 0.04% NaN_3_, 25 μM CMP-SA-PEO_4_-biotin) were subsequently added to each well. The sialylation reaction was initiated by the addition of 25 μL of the enzyme/sample dilutions to the plate. Plates were then sealed and incubated overnight at room temperature with gentle shaking.

The reaction was terminated by washing five times with excess PBST. 50 μL of a 1 μg/mL solution of Europium-labeled streptavidin (Perkin-Elmer) in PBST were then added to each well and incubated for approximately 1 h at room temperature with shaking. After washing five times with excess PBST, 100 μL of Enhancement Solution (Perkin-Elmer) were added to each well and incubated for 10 min at room temperature with shaking. Sialylation was detected by europium-based time-resolved fluorescence on a Beckman-Coulter DTX-880 plate reader.

Positive control ST6GalNAcI enzyme (chicken ST6 expressed and purified from Sf9 insect cell culture as described previously [25]) dilutions were prepared in dilution buffer (20 mM BisTris, 0.02% Tween-20, pH 6.7).

*E. coli *cell lysate samples were prepared by harvesting the cells corresponding to 1 mL of a culture with OD_600 _~ 2.0, resuspending these cells in 1 mL of dilution buffer, and pulse sonication on ice until cell lysis was achieved. The soluble cell lysate fraction was then acquired by high-speed centrifugation at 14,000 rpm for 20 min at 4°C and collection of the supernatant. Since the presence of more than 10% of cell lysate in the sialylation reaction mixture was found to inhibit the sialylation reaction, *E. coli *lysates were diluted 1:5 in dilution buffer prior to their addition to the wells containing reaction mixture. Positive control samples were corrected so as to include the same (10%) amount of bacterial cell lysate. One unit of ST6 activity was defined as the amount of enzyme that transferred 1 μmole of CMP-SA onto 1 mg of acceptor protein per minute at 25°C.

## List of Abbreviations

ST6: human sialyltransferase ST6GalNAcI; EPO: recombinant human erythropoietin; CMP-NeuAc or CMP-SA: cytidine monophosphate-N-acetylneuraminic acid; MBP: *E. coli *maltose-binding protein; Gal: β-D-galactopyranosyl; PEG: polyethylene glycol; IPTG: isopropyl-β-D-thiogalactopyranoside; BSM: bovine submaxillary mucin; aBSM: asialo (desialylated) BSM; PBS: phosphate-buffered saline; PBST: PBS + 0.l% Tween-20.

## Competing interests

The authors declare that they have no competing interests.

## Authors' contributions

GS, SDF and GG designed research; GS, SC, MFS and KMJ performed research and analyzed the data; GS and GG wrote the paper. All authors read and approved the final manuscript.
